# Veterinary Surgery of the War

**Published:** 1915-03

**Authors:** 


**Veterinary Surgery of the War.** To every division and cavalry brigade of the British expeditionary force, is attached a mobile veterinary section of one officer and 22 trained and mounted men of the Army Veterinary Corps. After first-aid treatment, horses are shipped by train to one of ten hospitals, each equipped to care for 1000 patients, a distinction being made between advanced and base hospitals. A convalescent depot covering 20 square miles has been established in France. Anaesthetics are regularly used for major operations. Aside from the humane element, the value of the horses saved for further service, is great. E. G. Fairholme, Sec. Royal S. P. C. A., 105 Jermyn St., London, in the Globe, quoted by Our Dumb Animals of Boston. The latter journal also states that Nebraska farmers have refused to sell horses to European agents.

